# Self-Schemas and Self-Esteem Discrepancies in Subclinical Paranoia: The Essential Role of Depressive Symptoms

**DOI:** 10.3389/fpsyt.2021.623755

**Published:** 2021-03-15

**Authors:** Manel Monsonet, Sergi Ballespí, Tamara Sheinbaum, Carmen Valiente, Regina Espinosa, Thomas Richard Kwapil, Neus Barrantes-Vidal

**Affiliations:** ^1^Departament de Psicologia Clínica i de la Salut, Universitat Autònoma de Barcelona, Cerdanyola del Vallès, Spain; ^2^Department of Psychology, University of Southern California, Los Angeles, CA, United States; ^3^School of Psychology, Complutense University of Madrid, Pozuelo de Alarcón, Spain; ^4^Department of Psychology, University of Camilo José Cela, Villanueva de la Cañada, Spain; ^5^Department of Psychology, University of Illinois at Urbana-Champaign, Champaign, IL, United States; ^6^Sant Pere Claver - Fundació Sanitària, Barcelona, Spain; ^7^Centre for Biomedical Research in Mental Health (CIBERSAM), Madrid, Spain

**Keywords:** paranoia, self-esteem, self-schemas, depressive symptoms, self-esteem discrepancies, implicit self-esteem

## Abstract

**Background :** Self-concepts are being intensively investigated in relation to paranoia, but research has shown some contradictory findings. Studying subclinical phenomena in a non-clinical population should allow for a clearer understanding given that clinical confounding factors are avoided. We explored self-esteem, self-schemas, and implicit/explicit self-esteem discrepancies in three non-clinical groups with different psychopathological traits and a control group.

**Methods:** Participants with elevated trait-paranoia (*n* = 41), depressive symptoms (*n* = 34), a combination of both traits (*n* = 32), and a control group (*n* = 71) were assessed on implicit and explicit self-esteem, self-schemas, depression, and paranoia. A dimensional approach with the total sample (*n* = 208) was also used to complement the information provided by the group approach.

**Results:** All groups presented similar and positive levels of implicit self-esteem. Trait-paranoia participants had similar levels of explicit self-esteem and self-schemas compared with the control group. However, the group with a combination of trait-paranoia and depressive symptoms showed the lowest levels of positive self-schemas and self-esteem. Furthermore, this group and the control group displayed implicit/explicit self-esteem discrepancies, although in opposite directions and with different implications. The dimensional approach revealed associations of trait-paranoia and depressive symptoms with poor explicit self-esteem and self-schemas but not with implicit self-esteem.

**Conclusions:** Trait-paranoia participants showed different self-representations depending on whether depressive symptoms were present or not. The interaction between subclinical neurotic and psychotic traits entailed a detrimental self-representation that might increase the risk for psychopathology.

## Introduction

Understanding psychopathological phenomena as a dimensional continuum, which range from mental health to severe mental illness, have a long tradition (1–5). Psychosis is currently conceptualized as a dynamic continuum that ranges from individual differences in schizotypy traits and subtle psychotic-like experiences through at-risk mental states (ARMS) or prodromal states to schizophrenia-spectrum disorders (6–8). Likewise, depression can be conceived as a dynamic continuum that extends from non-clinical experiences or feelings of depression to severe depressive disorders (9–11). Studying subclinical phenomena in non-clinical populations complements the information obtained from ill participants and provides a “cleaner” laboratory to grasp subtle psychological constructs by avoiding confusion factors such as elevated symptom severity, high comorbidity, medication side-effects, and the chronicity of the disorder itself, thus allowing to elucidate mechanisms of disorder risk, resilience, and onset (12).

Two psychological constructs that have attracted considerable attention are self-esteem (SE) and self-schemas (SC), which are widely involved not only in depression but also in psychosis, and more specifically, in paranoia (13, 14). Low SE is one of the diagnostic criteria for a DSM-5 diagnosis of major depressive disorder (15). However, it has also been demonstrated that neuroticism and low SE are risk factors for psychosis (16, 17). Besides, it is well-known that there is a high association between depression, low SE, negative SC, and persecutory delusions (18, 19), and it has even been suggested that some forms of paranoid schizophrenia might be camouflaged depression (20). Depression is also associated with severity, distress, prognosis, and relapse of psychotic symptoms (21), and it is estimated that comorbidity with schizophrenia occurs in 50% of patients (22). It has also been shown that negative SC (in combination with anxiety and negative-other self-evaluations), but not positive SC or low SE, were associated with non-clinical paranoia (23). However, other studies have found that low positive SC (24, 25) and low SE (26–28) correlated with paranoia in non-clinical populations. Thus, the precise pattern of associations between SC, SE, and paranoia in non-clinical populations remains unclear.

Regarding persecutory delusions, Bentall et al. (29) proposed a model based on a cycle of mutual influences between causal attributions and self-representations. They argued that in order to maintain deactivated implicit negative beliefs about the self, people with persecutory delusions attribute negative events to external agents. Although this “defensive model” did not intend to make any prediction about the role of explicit SE, some studies have ruled it out because low levels of explicit SE in paranoid patients have been documented. This defensive model only assumed, and showed evidence from implicit measures, that paranoid patients, like depressed people, have latent negative beliefs about the self. Nevertheless, recent findings are inconsistent with this assumption regarding paranoia, showing mixed results [see reviews: (18, 30, 31)]. In contrast, there is mounting evidence indicating that depressed patients show positive implicit SE, actually similar to that of healthy controls (32–35). One study assessed implicit SE using three different paradigms and found positive implicit SE for depressed participants in the three measures (32). Indeed, it has been demonstrated that high implicit SE is not necessarily advantageous for psychological health, and that discrepancies between explicit SE and implicit SE could be more damaging than high or low SE *per se* (36).

The study of discrepancies between explicit SE and implicit SE seems critical and intimately connected with paranoia, but the evidence is scarce (37). Discrepancies between explicit SE and implicit SE can occur in two different ways: higher explicit SE than implicit SE or higher implicit SE than explicit SE (36, 38), and it has been found that SE discrepancies in either direction could be damaging for psychological health (36, 37). As Bentall and colleagues proposed (29), measuring discrepancies between implicit SE and explicit SE might be especially relevant for testing the defensive model of paranoia. It could be expected that paranoid patients would have lower implicit SE than explicit SE. In addition, comparing levels of implicit SE between patients with persecutory delusions and patients without paranoia or healthy controls should provide meaningful evidence for a defensive model. To date, research has yielded contradictory results in clinical populations, with some studies supporting or partially supporting the defensive model (39–41), and others not (42–45). However, two of the three studies that assessed discrepancies directly comparing *z-*scores of implicit SE and explicit SE within groups of paranoid and depressed patients, and healthy controls reported no SE discrepancies in paranoid patients. Curiously, though, Kesting and colleagues (42) found differences between levels of explicit SE and implicit SE in healthy controls and depressed patients but not in acute and remitted deluded patients. Vázquez et al. (45) found similar results using identical groups but a different measure of implicit SE. In both studies, the depressed group showed the same pattern of discrepancy: higher implicit SE than explicit SE. Conversely, higher explicit SE than implicit SE was found in the healthy control group. Thus, it remains unclear whether differences between explicit SE and implicit SE characterize clinical and subclinical paranoid samples. To the best of our knowledge, no studies have tested SE discrepancies in non-clinical paranoia by directly comparing *z-*scores of implicit SE and explicit SE. Therefore, the present study was the first to examine SE discrepancies in subclinical paranoia. Moreover, it is the first time that levels of explicit SE and implicit SE, as well as SC, were compared among three non-clinical groups with elevations in different psychopathological traits: paranoia, depression, and a combination of the two.

The first goal of the present study was to examine levels and differential patterns of implicit SE and explicit SE, as well as SC, in non-clinical subjects with elevated levels of trait-paranoia with and without elevated levels of depressive symptoms, non-clinical subjects with high levels of depressive symptoms, and a control group. Secondly, discrepancies between *z*-scores of implicit SE and explicit SE were also explored within these four groups. Finally, a dimensional approach using the total sample (*n* = 208) was employed to explore whether trait-paranoia, depressive symptoms, and their interaction predicted SE, SC, positive discrepant SE (i.e., higher implicit SE than explicit SE), and negative discrepant SE (i.e., higher explicit SE than implicit SE).

Firstly, and according to Bentall et al. (29), it was hypothesized that trait-paranoia would be associated with negative implicit SE, whereas in line with recent research, depressive symptoms would be related to positive implicit SE. It was also predicted that subclinical paranoia and depression would be associated with low levels of explicit SE and SC. Specifically, we expected to find lower levels of explicit SE and SC in groups with elevated levels of depressive symptoms. Secondly, regarding the discrepancies between *z*-scores of explicit SE and implicit SE within groups, no discrepancies were expected in the trait-paranoia group. However, positive discrepant SE was predicted for the groups with depressive symptoms, while negative discrepant SE was expected for the control group. Finally, when using dimensional scores in the total sample, we hypothesized that trait-paranoia and depressive symptoms would be associated with explicit SE and SC, although a larger association with depressive symptoms was expected. We also predicted that trait-paranoia would not be related to any form of discrepant SE, whereas depressive symptoms would be related to positive discrepant SE.

## Methods

### Participants

The present study is part of an ongoing longitudinal project examining risk and resilience for psychosis (BLISS) (46). At time one, an unselected sample of 589 undergraduates at the Universitat Autònoma de Barcelona was screened for schizotypy traits. Usable screening data was obtained from 547 participants (42 were excluded due to invalid protocols). At time two, a smaller sample was selected for intensive assessment and participants with high schizotypy scores were oversampled in order to ensure an adequate representation of schizotypy variance. A detailed description of the sample selection procedure has been provided elsewhere (46, 47). A total of 208 (out of 214) participants, which successfully completed the implicit self-esteem assessment as well as other measures described above, were included in this study. The mean age of this sample was 19.7 years (SD = 2.3) and 77.9% were women. Four different groups were also yielded:

*The Depression group (DepG)* included those 34 participants of the total sample (*n* = 206) who scored in the top quartile of the Beck Depression Inventory-II (BDI-II) (48) and had trait-paranoia levels below the top quartile (percentile 73) as measured by the Suspiciousness subscale (SPQ-S) of the Schizotypal Personality Questionnaire (SPQ) (49).

*The Paranoia group (ParG)* included those 41 participants of the total sample who scored in the top quartile of the SPQ-S and had levels of depressive symptoms below the top quartile as measured by the BDI-II.

*The Mixed group (MixG)* included those 32 participants of the total sample who scored in the top quartile on both trait-paranoia and depressive symptom measures (SPQ-S and BDI-II, respectively).

*The Control group (ConG)* included those 71 participants of the total sample who scored below the percentile 50 on both measures.

### Materials and Procedure

The interviews were conducted by psychologists and advanced graduate students in clinical psychology who were trained in the administration of the measures and were unaware of participants' scores on the screening questionnaires. The study was approved by the Universitat Autònoma de Barcelona (Spain) Ethics Committee and conformed to the Helsinki Declaration.

#### Implicit SE

The Go/No-Go Association Task (GNAT) (50) was employed to assess implicit SE. The GNAT is conceptually based on the Implicit Association Test (IAT) (51), but it does not need the direct involvement of an opposed target category to make inferences (50, 52). Therefore, the GNAT has the advantage of analyzing automatic responses between the attribute concepts (e.g., positive, negative) and a single target category (e.g., the self). Williams and Kaufman (53) have demonstrated its reliability and some studies have shown its convergent, discriminant, and predictive validity (54, 55).

The GNAT self-esteem version used in this study was presented using Inquisit (Millisecond Software, 1996–2007). It comprised 28 words (of which 14 were positive and 14 negative) and assessed the strength of the automatic associations between words related to the concept of “Self” (e.g., myself, I, participant's first name) and positive attributes (e.g., smart, competent) and negative attributes (e.g., unable, stupid) (41). The 28 stimulus words were validated in a study of positive and negative adjectives related to self-worth (56). The GNAT had two critical blocks (self-positive and self-negative) randomly presented; each block contains the first 20 practice trials and then 60 critical trials. For each trial, one word appears in the middle of the screen while informative labels for the correct response are fixed in the upper left and right corners. Participants had to press the space bar only if the word that appears in the middle of the screen (e.g., smart) belongs to one of the two informative labels (e.g., self, positive). If the word did not match the informative labels (e.g., unable), participants did not have to respond. Words appear for a maximum of up to 1,200 ms or until the participant presses the space bar. Participants are instructed to respond as fast and accurately as possible and they had immediate feedback in each trail: A green *O* followed a correct response while a red *X* followed an incorrect response.

To calculate the implicit SE score, reaction times in the positive-self blocks were subtracted from reaction times in the negative-self blocks. A positive score means that the participant is faster associating the self with positive adjectives than the self with negative adjectives. This would be interpreted as positive implicit SE, whereas a negative score suggests negative implicit SE. Although other indices (e.g., *d'*) can be computed, reaction time indices have shown higher internal consistency (41) and much more internal reliability (50).

#### Explicit SE

To assess global explicit SE, a Spanish version (57) of the Rosenberg self-esteem scale (RSE) (58) was used. The RSE consists of five positively worded items and five negatively worded items (e.g., “I take a positive attitude toward myself”) measured on a four-point scale. Scores range from 0 to 30, where higher scores reflect higher explicit SE.

#### Discrepant SE

Following previous studies [e.g., (36, 37)], we created an index of discrepant SE. For each participant, standardized values of explicit SE were subtracted from standardized values of implicit SE. Then, in order to test whether trait-paranoia, depressive symptoms, and their interaction predicted a combination of higher levels of implicit SE than explicit SE (positive discrepant SE), or on the contrary, a combination of higher levels of explicit SE than implicit SE (negative discrepant SE), two continuous variables of discrepant SE were calculated. Positive discrepant SE variable included those participants with positive scores on discrepant SE (higher levels of implicit SE than explicit SE), whereas negative discrepant SE variable included those participants with negative scores on discrepant SE (higher levels of explicit SE than implicit SE). A score of 0 on discrepant SE (no differences between levels of implicit SE and explicit SE) indicated congruent SE.

#### Self-Schemas

The Brief Core Schema Scales (BCSS) is a self-reported measure that assesses beliefs about the self and others (23), which yields subscale scores for positive-self, negative-self, positive-others, and negative-others. For the purpose of this study, only positive-self and negative-self subscales were used. Each subscale contains six items rated on a five-point scale, ranging from 0 to 4, where lower scores mean low positive or negative SC, respectively.

#### Schizotypal Personality Questionnaire

The SPQ (49) is a self-reported scale to evaluate DSM-III-R schizotypal personality disorder. It consists of 70 dichotomous yes-no questions and yields nine subscales, one for each DSM schizotypal trait. The eight-item SPQ-S was used to measure trait-paranoia in this sample (e.g.: “I am sure I am being talked about behind my back”).

#### Beck Depression Inventory-II

The BDI-II (48) was used to dimensionally measure depressive symptoms. It contains 21 items that are rated on a four-point scale ranging from 0 to 3. Total score ranges between 0 and 63, where higher scores indicate greater severity of depressive symptoms.

### Statistical Analyses

All analyses were conducted using the Statistical Package for Social Sciences (SPSS), Version 19.0. Potential sex differences were tested with the Student's *t* test. To test for differences in sex and age among groups, a chi-square test and one-way ANOVA were respectively performed. Analyses of variance were used to compare groups on implicit SE, explicit SE, and discrepant SE and SC. To compare means, *post hoc* Tukey HSD or Tamhane tests were employed depending on the assumption of homogeneity of variances between groups. A mixed-model ANOVA with two within-subject variables (implicit SE and explicit SE) and one between-subject variable with four groups (DepG, ParG, MixG, and ConG) was conducted to test discrepancies between *z*-cores of implicit SE and explicit SE. Bonferroni correction for multiple comparisons was employed. Effects sizes were interpreted following Cohen (59): η^2^ = 0.01 as small, η^2^ = 0.06 as medium, and η^2^ = 0.14 as large. The strategy of comparing groups with “pure” trait-paranoia, depression, and mixed profiles allows for a comparison with clinical samples with depression and paranoia. At the same time, the continuous nature of measures and the non-clinical nature of the sample allowed for a complementary dimensional approach. Thus, a series of hierarchical linear regression were performed to explore the linear effect of paranoid traits, depressive symptoms, and their interaction term on the frequency of implicit SE, explicit SE, and discrepant SE and SC. Paranoia and depression scores were entered simultaneously at the first step to evaluate their unique contribution, and then the interaction term was entered at the second step to evaluate its effect beyond the main effects.

## Results

No sex differences were found for implicit SE (*t* = 0.996, *p* = 0.312), explicit SE (*t* = 0.132, *p* = 0.895), negative SC (*t* = −0.544, *p* = 0.587), positive SC (*t* = 0.694, *p* = 0.488), positive discrepant SE (*t* = 0.238, *p* = 0.813), negative discrepant SE (*t* = −0.169, *p* = 0.866), SPQ-S (*t* = −0.027, *p* = 0.978), and BDI-II (*t* = −0.141, *p* = 0.888) in the overall sample. Only a weak association between age and positive SC (*r* = −0.183, *p* = 0.008) was found. Regarding the GNAT, the means of the reaction times on the positive-self and negative-self blocks were 534.34 ms (range: 371.63–727.69) and 561.16 ms (range: 407.18–769.42), respectively, for the whole sample. Therefore, the mean of the implicit SE score was 26.82 ms (range: −137.80–136.67) for the whole sample. There were no significant differences among groups on age (*F*_[3,174]_ = 0.980, *p* = 0.404) or sex (χ^2^ = 2.396, *p* = 0.494). Descriptive data on paranoia and depression measures for all groups are presented in Table 1. As expected, the ParG and the MixG groups had higher levels of paranoia than the DepG and the ConG (*F*_[3,174]_ = 235.65, η^2^ = 0.80, *p* < 0.001). Similarly, the depression and the mixed groups had higher levels of depressive symptoms than the paranoia and the control groups (*F*_[3,174]_ = 129.91, η^2^ = 0.69, *p* < 0.001).

**Table 1:** Means (standard deviations) and score ranges on paranoia and depression measures for groups.

	**BDI-II**	**SPQ-S**
Control group	1.34 (1.16); range: 0–3	1.14 (0.76); range: 0–2
Depression group	11.56 (5.17); range: 7–29	2.26 (1.26); range: 0–3
Paranoia group	3.24 (2.05); range: 0–6	4.90 (1.01); range: 4–8
Mixed group	11.59 (4.35); range: 7–25	5.88 (1.04); range: 5–8

*BDI-II, The Beck Depression Inventory-II; SPQ-S, The Suspiciousness subscale of the Schizotypal Personality Questionnaire (SPQ)*.

Firstly, the results of ANOVAs comparing implicit SE and explicit SE, discrepant SE, as well as SC across groups are presented in Table 2. No significant differences were found among groups for implicit SE (*F*_[3,174]_, η^2^ < 0.01); all groups displayed positive implicit SE. Conversely, there was a large group effect for explicit SE (*F*_[3,171]_, η^2^ = 0.37). *Post hoc* tests indicated that the MixG had lower levels of explicit SE than the other groups (ConG and ParG: *p* < 0.001; DepG: *p* = 0.033), whereas the DepG showed lower levels than the ParG (*p* = 0.011) and the ConG (*p* < 0.001). Thus, the paranoia and control groups had similar levels of explicit SE (*p* = 0.306). There was also a large group effect for negative SC (*F*_[3,174]_, η^2^ = 0.17), with the MixG showing higher negative SC than the ParG and ConG (all *p*-values <0.005). Positive SC was also significantly different across groups (*F*_[3,174]_, η^2^ = 0.08). The MixG had a lower positive SC than the ConG (*p* = 0.002). Thus, the depression, paranoia, and control groups showed no significant differences for positive and negative SC. There was a large group effect for positive discrepant SE (*F*_[3,74]_, η^2^ = 0.15), indicating that the MixG had a higher positive discrepancy than the ConG. A medium group effect for negative discrepant SE (*F*_[3,93]_, η^2^ = 0.09) was also found, with the ParG showing a higher negative discrepancy than the MixG. No other differences were found among groups.

**Table 2:** Mean differences (and standard deviations) in self-esteem and self-schema variables among groups.

**Groups**	**CG**	**DG**	**PG**	**MG**	**ANOVA overall**	***p***
	**(*n* = 71)**	**(*n* = 34)**	**(*n* = 41)**	**(*n* = 32)**		
Implicit SE	25.81 (42.54)	22.03 (37.68)	23.90 (54.74)	19.89 (49.08)	*F* = 0.14	0.937
Explicit SE	25.50 (2.94)	21.03 (4.16)	24.18 (4.07)	17.78 (4.92)	*F* = 33.37	<0.001
Summary of *post hoc* tests: CG, DG, PG > MG; CG, PA > DG						
Negative SC	1.65 (1.94)	3.50 (4.16)	2.34 (2.09)	5.03 (3.60)	*F* = 11.46	<0.001
Summary of *post hoc* tests: MG > CG, PG						
Positive SC	13.72 (4.78)	11.85 (3.93)	12.76 (4.87)	10.06 (4.37)	*F* = 4.97	0.002
Summary of *post hoc* tests: MG < CG						
Positive discrepant SE^*^	(*n* = 19)	(*n* = 19)	(*n* = 18)	(*n* = 22)		
	0.77 (0.47)	1.28 (0.81)	1.17 (0.90)	1.76 (1.20)	*F* = 4.19	0.009
Summary of *post hoc* tests: MG > CG						
Negative discrepant SE^*^	(*n* = 50)	(*n* = 14)	(*n* = 22)	(*n* = 10)		
	−1.02 (0.68)	−0.91 (1.01)	−1.47 (0.95)	−0.69 (0.81)	*F* = 3.06	0.033
Summary of *post hoc* tests: PG > MG						

*CG, control group; DG, depression group; PG, paranoia group; MG, mixed group; ANOVA, analysis of variance; SE, self-esteem; SC, self-schemas*.

* *n indicates the number of participants within each group assigned to positive or negative SE discrepancy*.

**Figure 1:**
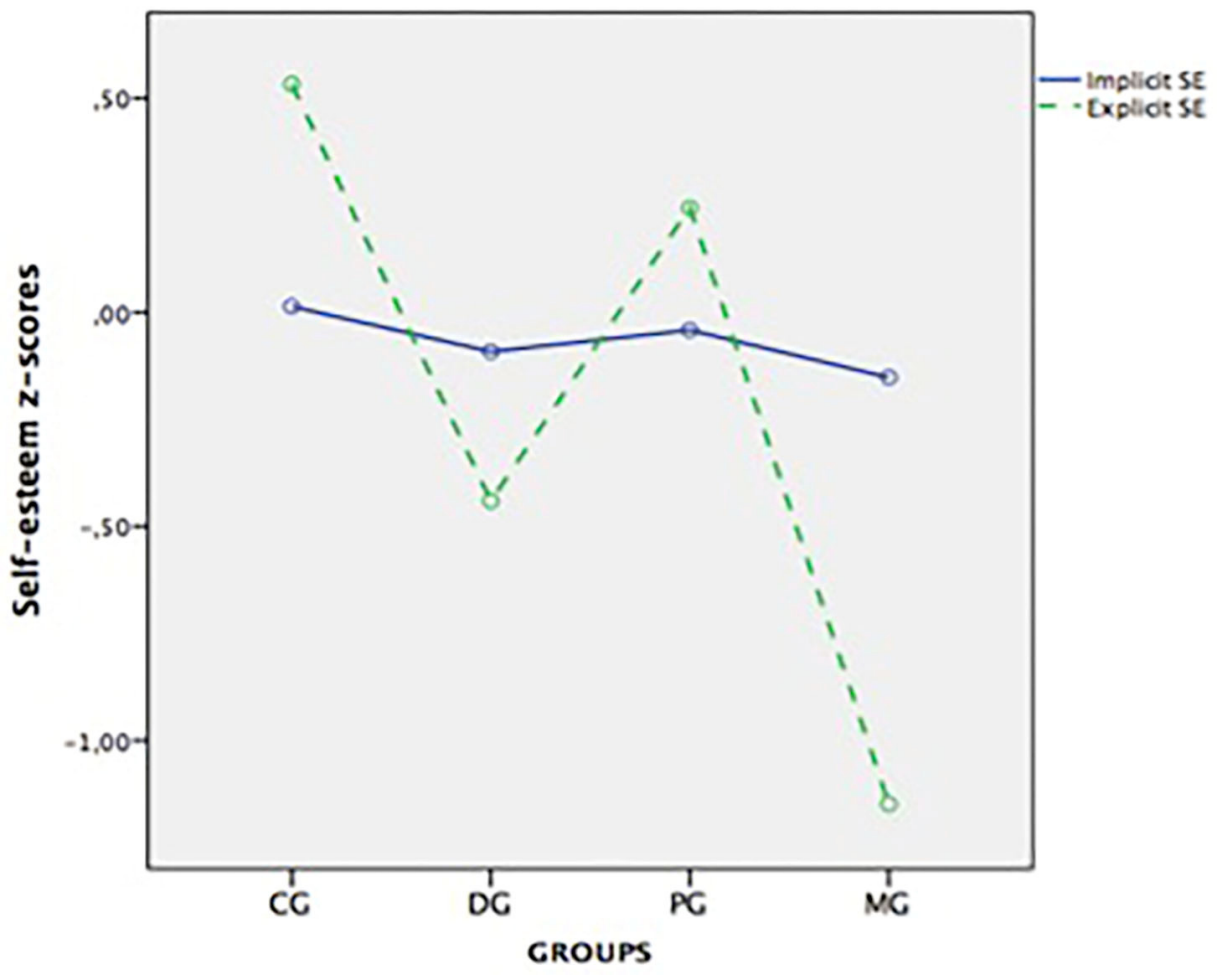
Means of explicit and implicit self-esteem *z*-scores in the control (CG), depressive (DG), paranoia (PG), and mixed (MG) groups.

Secondly, the analysis of differences between *z*-cores of implicit SE and explicit SE revealed that the main effect was not significant (*F* = 1.61, *p* = 0.207, η^2^ = 0.00), whereas a significant effect for group was found (*F* = 17.93, *p* < 0.001, η^2^ = 0.24). The ConG had a higher overall SE than the DepG and the MixG, while the ParG showed higher SE than the MixG (all *p*-values <0.001). A significant two-way interaction between SE and group was also obtained (*F* = 10.64, *p* < 0.001, η^2^ = 0.16). Within-group pairwise comparisons revealed a discrepancy between explicit SE and implicit SE in the ConG (*p* < 0.001, η^2^ = 0.06) and the MixG (*p* < 0.001, η^2^ = 0.09). The ConG had higher explicit SE than implicit SE, whereas the opposite pattern was found in the MG. The depression and paranoia groups did not show a statistically significant discrepancy (*p* = 0.138, η^2^ = 0.01; *p* = 0.182, η^2^ = 0.01; respectively). Between-group pairwise comparisons for explicit SE and implicit SE revealed similar results to those described above with one-way ANOVA analyses. Means of implicit SE and explicit SE *z*-scores for all groups are depicted in Figure 1.

Finally, the results of the regression models conducted with the total sample (i.e., dimensional approach) are presented in Table 3. Of the total sample (*n* = 208), three participants had RSE invalid data; therefore, for explicit SE and discrepant SE variables, the total sample was 205 participants. Of these, 96 had positive discrepant SE (higher levels of implicit SE than explicit SE), 108 had negative discrepant SE (higher levels of explicit SE than implicit SE), and one participant had congruent SE (no differences among implicit SE and explicit SE). For each predictor (trait-paranoia, depressive symptoms, and their interaction), the standardized regression coefficients (β), semipartial *r*^2^, and effect sizes *f* ^2^ are reported. Trait-paranoia predicted explicit SE and negative SC, while a trend was observed for positive SC (*p* = 0.059). In contrast, trait-paranoia was not associated with implicit SE or with any type of discrepant SE. Depressive symptoms were negatively associated with explicit SE and positive SC but positively associated with negative SC and positive and negative discrepant SE. Conversely, depressive symptoms did not predict implicit SE. Lastly, the interaction term between trait-paranoia and depressive symptoms predicted levels of explicit SE beyond the main effects (and a trend was observed for negative SC; *p* = 0.057). No more significant associations were found.

**Table 3:** Linear regressions of self-esteem and self-schemas measures (*n* = 208).

	**Step 1 (*****df*** **=** **205)**						**Step 2 (*****df*** **=** **204)**		
**Criterion**	**Trait-paranoia**			**Depressive symptoms**			**Interaction**		
	**β**	**Δ*r*^**2**^**	***f*^**2**^**	**β**	**Δ*r*^**2**^**	***f*^**2**^**	**β**	**Δ*r*^**2**^**	***f*^**2**^**
Negative SC	0.191^**^	0.095	0.04	0.312^***^	0.147	0.10	0.125^†^	0.055	0.02
Positive SC	−0.137^†^	0.045	0.02	−0.204^**^	0.065	0.04	−0.050	0.016	0.00
Implicit SE	−0.025	0.001	0.00	−0.020	0.001	0.00	−0.037	0.002	0.00
*n* = 205	Step 1 (*df* = 202)						Step 2 (*df* = 201)		
Explicit SE	−0.142^*^	0.130	0.03	−0.574^***^	0.394	0.48	−0.112^*^	0.080	0.02
Positive discrepant SE	0.144	0.066	0.02	0.356^**^	0.162	0.14	0.131	0.086	0.02
Negative discrepant SE	−0.164	0.006	0.02	0.226^*^	0.027	0.05	0.058	0.009	0.00

† *p < 0.06*;

* *p < 0.05*;

** *p < 0.01*;

*** *p < 0.001*.

*SE, self-esteem; SC, self-schemas*.

## Discussion

The present study compared three non-clinical groups with paranoia, depression symptoms, and a combination of the two features, in addition to a control group, to shed light on the association of these constructs with implicit SE, explicit SE, and discrepant SE and SC. In line with our hypotheses, we found no discrepancies between explicit SE and implicit SE in the trait-paranoia group; although explicit SE was slightly higher than implicit SE, both measures were positive and did not significantly differ. Likewise, dimensional analyses revealed that neither trait-paranoia nor the interaction between trait-paranoia and depressive symptoms were associated with implicit SE, thus replicating and expanding the results found by Cicero and Kerns (26) who found that implicit SE was unassociated with paranoia in non-clinical participants using a different measure of implicit SE. Finally, positive and similar levels of implicit SE were found across all groups. Therefore, these findings do not support Bentall's defensive model in a non-clinical population with trait-paranoia, regardless of whether depressive symptoms were present or not. It is likely that some phenomena in the psychotic spectrum may differ in their expression depending on the severity of the associated impairment. Thus, it could be argued that while subclinical paranoid experiences do not reach the severity of persecutory delusions implicit SE would remain preserved and psychotic defenses would not be triggered. However, two of three studies with paranoid patients that directly compared *z*-cores of implicit SE and explicit SE (42, 45) also found no discrepancies in the paranoid group. Furthermore, the fact that these studies, and the present study, employed different paradigms to assess implicit SE seems to strengthen the hypothesis that paranoia is not associated with SE discrepancies or low implicit SE. Alternatively, it has been suggested that SE discrepancies would differ depending on the person's belief about the deservedness of the persecutory delusion. Whereas, “bad-me” paranoia patients believe that the persecution is deserved, basically because the self is viewed as bad, “poor-me” paranoia patients believe that the persecution is not deserved (60). Nakamura and collaborators (40) found that poor-me paranoia patients, but not Bad-me, showed a SE discrepancy. However, this finding should be interpreted with caution given the small sample size recruited of the poor-me paranoia group (*n* = 14) and the fact that when both paranoid groups were jointly assessed (*n* = 35), no SE discrepancy was found. Actually, it has been claimed that the concept of deservedness in paranoia is not a specific categorical distinction (61), but rather a dimensional facet of paranoia (62).

As expected, the control group presented a statistically significant discrepancy, with higher levels of explicit than implicit SE. Nevertheless, both measures of SE were positive, reflecting optimal levels of implicit SE and explicit SE. Thus, it could be inferred that the control group had secure SE, as there is no need to adopt defensive strategies to protect the self from eventual hazards (63). Kesting et al. (42) speculated that representing oneself explicitly in a more positive way than oneself implicitly feels might be protective. Then, this pattern of self-representation would be adaptive or normative to healthy people reflecting normal cognitive processes. However, it might also simply reflect a social desirability bias when responding to personality or attitudinal questionnaires (64). In contrast, the group with depressive symptoms showed higher implicit SE than explicit SE; however, this difference was not statistically significant, contrary to what was found in clinical samples (42, 45). Probably, the fact that our participants did not have clinical depression and, therefore, their levels of explicit SE remained relatively preserved may explain the differences in SE discrepancies found among clinical and non-clinical samples with depressive symptoms. Nevertheless, in line with other studies (32, 34, 35, 65) and in accordance with our hypothesis, the depression group showed positive or normal levels of implicit SE. Finally, the mixed group did have a significant positive discrepancy (as expected for the depression group), showing low explicit SE and positive implicit SE. Thus, the mixed group was the only group that presented insecure SE (36), specifically, damaged SE (66). However, this pattern of unbalanced self-representation where implicit SE was normal or positive and explicit SE was low does not match with the pattern of discrepancy predicted by Bentall and colleagues in paranoia patients (29). In sum, our participants with a combination of depressive symptoms and trait-paranoia showed a similar pattern to that of clinically depressed patients (33, 41, 42, 45) and indicate different self-representations in trait-paranoia individuals depending on whether depressive symptoms were present or not. This emphasizes the relevance of taking into account the interaction between depressive and paranoid symptoms and suggests that this interaction should be considered regarding the etiology and prognosis of paranoid ideation. Notably, patients with schizophrenia who had suffered depression at the prodromal stage exhibited more severe first psychotic episodes and more depressive and positive symptoms over the initial 5-year course (67).

Regarding explicit SE, our hypotheses were partially supported, as the depression and mixed groups showed the lowest levels. In contrast, the paranoia group had similar levels of explicit SE to the control group. Although recent reviews (18, 31) indicated an association of low explicit SE with paranoia in patients and non-clinical populations, some studies did not control for depressive symptoms while others found positive or normal levels of SE in paranoid patients (41, 60, 68). Furthermore, one study with a large sample size also found no differences in explicit SE between high and low non-clinical paranoid participants (27). It might be that low levels of explicit SE in paranoia were mainly dependent on the neurotic processes that, as many studies pointed out [e.g., (69, 70)], are frequently involved in the formation and maintenance of psychotic delusions.

A slightly different picture appears concerning positive and negative SC. The depression and paranoia groups had slightly low levels of positive SC and high levels of negative SC than the control group; however, this difference did not reach statistical significance. Thus, contrary to our expectations, the depression and paranoia groups showed similar levels of SC to the control group. This seems to indicate that as long as paranoia traits and depressive symptoms do not exceed the clinical threshold, SC will remain relatively preserved. Our results are in agreement with Taylor et al. (71), who found very similar levels of SC in non-clinical participants who endorsed some schizotypy experiences, and with Espinosa and colleagues (72), who reported similar levels of negative beliefs about the self between depressed and paranoid patients. Conversely, levels of positive and negative SC in the group with a combination of trait-paranoia and depressive symptoms were lower than in the control group and comparable with those found in individuals at clinical high risk of developing psychosis (71, 73, 74). Therefore, once again, the mixed group displayed an unfavorable conscious self-representation that seems relevant for both the negative and the positive dimensions of SC.

Finally, whereas the dimensional approach with the total sample revealed significant associations of trait-paranoia with explicit SE and negative SC (only a trend was present for positive SC), depressive symptoms were related to explicit SE, positive and negative SC, and SE discrepancies. The fact that in the dimensional approach, where the whole sample was examined, trait-paranoia was not associated with any type of discrepant SE is in line with the finding that the paranoia group did not show a discrepancy between explicit SE and implicit SE. Instead, depressive symptoms in the total sample were related to both positive and negative discrepant SE, although the association of depressive symptoms with the positive discrepancy was larger, which is in line with findings in clinical depression (33, 42, 45). Participants with negative discrepant SE would be more susceptible to have fluctuating SE discrepancies because having low implicit SE and high explicit SE is associated with an unstable explicit SE (75), which can partly explain the association of depressive symptoms with negative discrepant SE. It is also noteworthy that the interaction term negatively predicted explicit SE beyond the main effects of trait-paranoia and depressive symptom, and was very close to statistical significance in negative SC. This seems to confirm, also in the total sample, that the interaction between trait-paranoia and depressive symptoms aggravates the negative self-concept of individuals, which might increase the risk for psychopathology. The association of depressive symptoms with explicit SE was of a large magnitude, and very close to a medium with positive discrepant SE, whereas the other effect sizes were small. Numerous studies in clinical and non-clinical populations also found significant associations of SE and SC with paranoia but, as this study pointed out, depressive symptoms boosted these associations, and effect sizes must be reported to quantify the magnitude of the relationship (76, 77). Only then a clearer picture of the association between self-representation and paranoia can be obtained.

Concerning the limitations of this study, its cross-sectional nature prevents drawing conclusions about causal effects. Furthermore, given that the participants are pursuing a Psychology degree, it is possible that their psychological knowledge and the awareness of some phenomena may bias their reports and also limits the generalizability of the findings to community samples. Another limitation of the study is that an *a priori* power analysis to determine sample sizes was not performed. Finally, it has been argued that implicit SE is only an impoverished measure of explicit SE and it should not be labeled as such (78). Although the GNAT overcame some of the weaknesses of previous measures of implicit SE, the precise nature of the unconscious self-related association that the GNAT, or other implicit SE tasks, assesses is still a matter of debate. Further research is needed for a better understanding of the measures that seek to delve into the non-conscious psychological processes. Similarly, further studies should be carried out in order to gain more in-depth knowledge about SE discrepancies in clinical and subclinical paranoia, as reducing maladaptive SE discrepancies could be an effective strategy to reduce paranoid ideation (37, 79).

The present study showed that levels of positive and negative SC, explicit SE, and SE discrepancies in subclinical paranoia depend on the presence and levels of depressive symptoms. In contrast, positive and similar levels of implicit SE were found in subclinical paranoia regardless of the presence of depressive symptoms. In line with recent research, our findings indicate that paranoia was related to negative explicit SE, but no association was found between paranoia and implicit SE (30, 43). Thus, individuals with subclinical paranoia would present different self-representations depending on whether depressive symptoms were present or not. The combination of trait-paranoia and depressive symptoms yielded an unfavorable conscious self-concept, suggesting that the interaction between subclinical neurotic and psychotic traits entailed a detrimental self-representation that could entail an increased risk for the development of psychopathology.

### Ethical Standards

The authors assert that all procedures contributing to this work comply with the ethical standards of the relevant national and institutional committees on human experimentation and with the Helsinki Declaration of 1975, as revised in 2008. All participants provided an informed consent.

## Data Availability Statement

The raw data supporting the conclusions of this article will be made available by the authors, without undue reservation.

## Ethics Statement

The studies involving human participants were reviewed and approved by the Ethics Committee of the Universitat Autònoma de Barcelona (Comissió d'Ética en l'Experimentació Animal i Humana (CEEAH); number 701H-JS). The patients/participants provided their written informed consent to participate in this study.

## Author Contributions

MM, SB, and NB-V conceived the study. SB, TS, and NB-V interviewed participants and collected data. MM and TK analyzed the data. MM, TK, and NB-V wrote the paper. TS, CV, and RE critically revised the manuscript. All authors contributed to the article and approved the submitted version.

## Conflict of Interest

The authors declare that the research was conducted in the absence of any commercial or financial relationships that could be construed as a potential conflict of interest.
